# Influence of Maternal Exposure to Mass Media on Growth Stunting Among Children Under Five: Mediation Analysis Through the Water, Sanitation, and Hygiene Program

**DOI:** 10.2196/33394

**Published:** 2022-04-06

**Authors:** Shutong Huo, Kai Wang, Zongchao Liu, Yuao Yang, Jia Yi Hee, Qiwei He, Rie Takesue, Kun Tang

**Affiliations:** 1 Vanke School of Public Health Tsinghua University Beijing China; 2 Program in Public Health University of California Irvine, CA United States; 3 Department of Biostatistics Mailman School of Public Health Columbia University New York, NY United States; 4 Health Section Programme Division UNICEF Headquarters New York, NY United States; 5 Institute of Healthy China Tsinghua University Beijing China

**Keywords:** water, sanitation and hygiene, mass media, malnutrition, Democratic Republic of Congo, DRC, mediation analysis, children, pediatric, stunting, television, internet, sanitation, hygiene

## Abstract

**Background:**

The issue of malnutrition in the Democratic Republic of Congo is severe. Meanwhile, the Water, Sanitation, and Hygiene program has been demonstrated to be effective in reducing the rates of growth stunting among children.

**Objective:**

We aimed to explore the association between maternal exposure to mass media and stunting in children through water, sanitation, and hygiene behaviors.

**Methods:**

Mediation analysis was conducted using data from the 2018 Multiple Indicators Cluster Surveys.

**Results:**

Mothers’ exposures to television and the internet in the Democratic Republic of Congo significantly decreases the risk of stunting in children by 5% and 10%, respectively, mediated by household water, sanitation, and hygiene facilities and practices.

**Conclusions:**

These findings could inform interventions and policies to reduce the rate of stunting rate children by promoting water, sanitation, and hygiene through mass media, especially through the internet and television.

## Introduction

Undernutrition resulted in approximately 45% of deaths in children under 5 years, while stunting affected approximately 149 million children under 5 years in 2020 globally [1], posing significant global health issues. The United Nations Children's Fund (UNICEF) defines *stunting rate* as the percentage of children aged between 0 to 59 months whose height is between 2 standard deviations (moderate and severe stunting) and 3 standard deviations (severe stunting) below the median for their age [2]. World Health Organization (WHO) data indicate that sub-Saharan Africa has the highest prevalence of stunting globally—approximately 32.3% children under 5 years in 2020 [3]. In sub-Saharan Africa, the largest percentage of children with stunted growth, approximately 43% in 2010, were from the Democratic Republic of Congo [2]. This issue gained the attention of the World Health Assembly, and 6 global nutrition targets were set for 2025 [3]; in 2012, the first target—a 40% reduction in the number of children under 5 years with stunted growth—was met. However, in 2017, 42% of children in the Democratic Republic of Congo had stunted growth [4]. The prevalence of stunting continues to increase in the Democratic Republic of Congo, despite actions being implemented to reduce stunting in children in the Democratic Republic of Congo [4,5]. Stunting has been demonstrated to negatively affect cognitive performance, educational performance, and maternal reproductive outcomes.

WHO identified poor maternal health and nutrition, inadequate infant- and child-feeding practices, and infections as the main causes of stunting in children under 5 years. In addition, unsafe water sources and poor handwashing practices are also major factors that cause stunting [3,6]. Safe water sources and improved hygiene through good handwashing practices are the primary components of the WHO Water, Sanitation, and Hygiene (WASH) program, which is a primary health service initiative to provide access to healthy and safe water, and sanitation facilities including soap and water for proper handwashing [7]. By building community-based interventions, such as the WHO WASH program, children will be better guarded against diarrheal diseases, malaria, intestinal worms, and environmental causes of subclinical infection, and prevention of these illnesses will lower the risk of stunting in children [3]. Several experimental studies [8-13] have demonstrated that increased access to water, sanitation, and hygiene is significantly associated with lower risks of stunting. A randomized experiment in India reported that the implementation of a sanitation program resulted in reduced stunting [8], and a cluster-randomized controlled trial in rural Mali found that sanitation intervention using behavioral modification gradually increased the availability of and accessibility to latrines, and reduced stunting in children [9]. Furthermore, a number of programs have been executed globally, such as the US Centers for Disease Control and Prevention Global Water, Sanitation, and Hygiene program, which resulted in a 25% decrease in childhood diarrhea, and the UNICEF WASH Program, which has provided access to healthy water to 14 million people for daily use in cooking, drinking, and personal hygiene [14-16].

Because only approximately 50% of the population in the Democratic Republic of Congo have access to clean water sources, UNICEF supplies safe drinking water, maintains sufficient sanitation facilities, and promotes hygienic practices during critical periods in communities and schools [17]. In the Democratic Republic of Congo, several water, sanitation, and hygiene awareness-raising sessions have been organized with campaigners who disseminate information by directly addressing the public or through mass media (eg, radio, television, and the internet) [2,18,19]. Regionally, the Democratic Republic of Congo WASH Consortium Program improved household health and reduced water-associated illnesses in 2020 [20]. The program used social media, such as Twitter and Facebook, radio broadcasts, and newsletters to disseminate knowledge and evidence about water, sanitation, and hygiene [21]. Literature has shown that mass media directly influences people's behavior, and the effect increases quickly by improving mass media [22,23]. In addition, studies [24-26] conducted in other countries have demonstrated that media access is associated with better water, sanitation, and hygiene behaviors, especially in resource-poor settings. The use of mass media has also been associated with reduced rates of stunting. In sub-Saharan Africa, stunting is significantly associated with mass media exposure [27-29]. Studies conducted in other low- or middle-income countries, such as Bangladesh [30], Indonesia [31], and India [32] also found that the exposure of mother to the mass media is associated with stunting. In India, a community-led and community-managed programs using technology and mass media to change community behavior reduced stunting rates in children under 2 years [3].

Although published research has explored whether water, sanitation, and hygiene facilities and practices are associated with stunting; whether mass media are associated with water, sanitation, and hygiene facilities and practices; and whether mass media are associated with stunting; there is little research on associations between mass media, stunting, and water, sanitation, and hygiene–related behaviors as an integral. Hence, we aimed to investigate whether maternal exposure to mass media influences stunting rates and water, sanitation, and hygiene facilities and practices and to identify which type of mass media exposure works best to improve water, sanitation, and hygiene and nutrition behaviors.

## Methods

### Data Source and Study Design

Data from the UNICEF Multiple Indicator Cluster Surveys (MICS) were utilized. MICS, which consists of 6 rounds of surveys that focus mainly on maternal health and child development, has become the largest source of statistically sound and internationally comparable data on women and children worldwide [33]. Ethics approval for the survey was provided by individual review boards within each participating country at the time of survey implementation. Surveys were conducted in 26 provinces throughout urban and rural areas in the Democratic Republic of Congo during 2017 to 2018, in which a multistage clustering sampling strategy was used to select households. Type of residence was retained for stratification. With sampling units at different levels, a total of 21,630 households were drawn. From the original data, a sub–data set was formalized, in which 20,019 households remained after matching for household indices across 3 correlated data sets. The sub–data set documented basic and health-related information for women of reproductive age as well as their children under the age of 5 years.

### Measures and Outcomes

#### Basic Characteristics

Data on maternal education level, household wealth, child's sex and age, residency, and Democratic Republic of Congo province of residence were utilized for this study. *Maternal education level* was coded as an ordinal discrete variable with 3 categories (no formal education or did not complete primary school, completed primary school, completed secondary school). *Household wealth* was categorized by quintiles of wealth index (low, low-middle, middle, high-middle, and high). *Child's age* in years was coded as an ordinal categorical variable ranging from 0 to 4. *Sex* (male or female) and *residency* (urban or rural) were both binary variables, and *province* of residence was coded as a nominal categorical variable (26 provinces).

#### Primary Outcome

The primary outcome of this study was whether a child in a given household was considered stunted, characterized with the binary variable *stunting*.

#### Primary Exposure

The primary exposure of this study was maternal exposure to mass media. Exposure included variables that indicated whether a mother was exposed to magazines or newspapers, radio, television, and the internet. As families in Democratic Republic of Congo often have limited access to these items, these variables were coded as binary variables rather than with multilevel values indicating detailed frequencies.

#### Mediator

The potential mediator of this study was coded as a composite variable that summarized information on household water, sanitation, and hygiene facilities and practices. Data from questions in the survey were grouped into handwashing practice, appropriate point-of-use water treatment, improved toilet, and improved drinking water source. These questions were addressed in our study either by household‐level spot check observations or by self‐reporting. After filtering valid responses, data from the 4 items were summarized into one composite variable, with 3 ordinal levels. Level 1 indicated that the household met none of the criteria, level 2 indicated that the household met at least one but not all of the criteria, and level 3 indicated that all of the criteria were met. A higher level indicated better water, sanitation, and hygiene facilities and practices.

Among 20,019 households in the sub–data set, 19,397 were included in the analysis based on responses to the questions and child's age in the household. Given the limited information about water, sanitation, and hygiene facilities and practices covered by the survey and conceiving reasonable associations among the primary outcome, exposure, and the potential mediator, only a subset of the original survey questions was taken into account.

### Statistical Analysis

**Figure 1:**
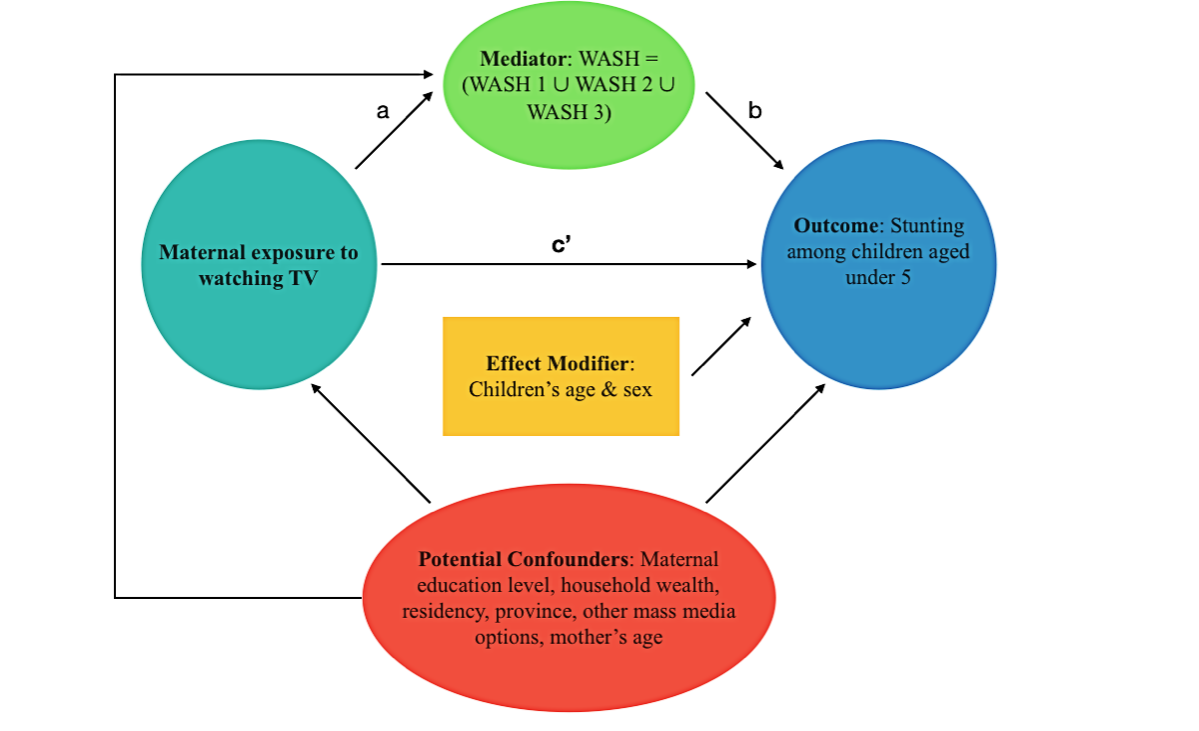
Potential mediation effects in causal pathways. a and b: average causal mediation effect (ACME); c': average direct effect (ADE); WASH: Water, Sanitation, and Hygiene.

Descriptive and regression analyses were performed to investigate the causal relationships between water, sanitation, and hygiene; stunting; and mother's exposure to mass media. Descriptive analysis was conducted on baseline characteristics, primary outcome and exposure, and potential mediator. Categorical variables were described as proportions, and continuous variables were described with mean with standard deviation. Mediation analysis (Figure 1) was performed using binary logistic regression:



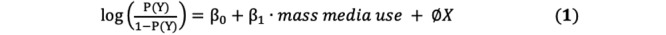



where *Y* denotes an event that whether the child is stunted as a binary outcome, *β*_1_ denotes the coefficient for mass media use, and *β*_0_ denotes the intercept of the fitted model, which also includes a vector of control variables *X* with their coefficients phi (or *Z* with their coefficients theta), and ordinal logistic regression,



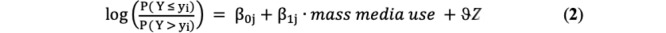



where *Y* denotes the water, sanitation, and hygiene practice composite variable with an ordinal outcome of *m* levels (1 ≤*i* ≤(*m*–1)), *β*_1_*_j_* denotes the coefficients for mass media use, *β*_0_*_j_* denotes the intercept of the fitted model, and the model also includes a set of control variables *Z*.

Model 1 was a logistic regression model that included the mass media use, maternal education level, child's age and sex, family wealth, residency, province, and mother's age as covariates and stunting as the outcome. Model 2 was an ordinal regression that included the mass media use, maternal education level, family wealth, residency, province, and mother's age as covariates and the composite variable as the outcome. The hypothesized mediating role of the composite variable between mass media exposure and stunting was tested for each media type. Combining model 1 and model 2 together,



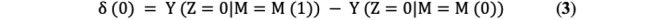





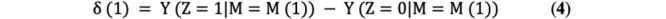



where *Y* denotes the stunting outcome, *Z* denotes the mass media use, *M* denotes the water, sanitation, and hygiene facilities and practices, and *δ* denotes the average causal mediation effect, which can be achieved by subtracting Equation 3 from Equation 4;



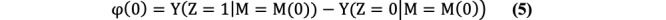





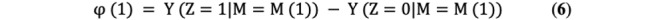



where *Y* denotes the stunting outcome, *Z* denotes the mass media use, *M* denotes the water, sanitation, and hygiene facilities and practices practice, and φ denotes the average direct effect, which can be achieved by subtracting Equation 5 from Equation 6; and total effect in the causal pathway was estimated using a general model-based approach [34].

Total effect = Average causal mediation effect + Average direct effect

All statistical analyses were performed using R (version 3.6.1). The level of statistical significance was set at 5% (*P*<.05) for all statistical tests. Results were reported as odds ratio (OR) and 95% confidence intervals. Interpretation for the coefficients of the ordinal regression model was slightly different from that of the logistic regression models, despite the same link function—a general model-based approach to mediation analysis [35,36] was used to examine the mediating effects, implemented using an R package [34]. The point estimates and 95% confidence intervals for average causal mediation effect, average direct effect, total effect, and the proportion of mediation were reported for each type of media. The number of Monte Carlo draws for quasi-Bayesian approximation was set to 1000. The White heteroskedasticity-consistent estimator for the covariance matrix was used to attain robust standard errors.

## Results

The prevalence of stunting was found to be 44.43% (8619/19,397) in children under 5 years (Table 1). The majority of children were from households without improved drinking water sources (12,288/19,397, 63.35%), appropriate water treatment (18,961/19,397, 97.75%), improved toilets (17,213/19,397, 88.74%), and appropriate handwashing practices (17,130/19,397, 88.31%). Most children resided in rural areas (14,191/19,397, 73.16%), and more than half of the mothers had primary education levels or less.

Radio had the highest access rate (3943/19,397, 20.33%) among all the mass media methods, followed by television (2300/19,397, 11.86%), magazine or newspaper (1084/19,397, 5.96%), and internet (342/19,397, 1.76%). In the descriptive analysis, compared to children of mothers who have never been exposed to mass media, children of mothers who have been exposed to mass media had a higher-value composite variable (ie, better water, sanitation, and hygiene practices), lower prevalence of stunting, higher household wealth level, higher mother's education level, and were more likely to reside in urban areas. Provincial information can be found in Multimedia Appendix 1.

Mothers’ exposure to television or internet significantly improved household water, sanitation, and hygiene practices, after adjusting for mother's education level, household wealth level, residency, province of residence, and mother's age (Table 2). For children whose mother had ever watched television, the odds of the combined high and middle water, sanitation, and hygiene practices versus that of the low level were estimated to be 1.64 times those of children whose mother had never watched television. For children whose mother had ever used the internet, the odds ratio of the same was estimated to be 2.89. The logistic model in which stunting was regressed on the composite variable showed a significantly decreased effect of water, sanitation, and hygiene practices variable on the odds of stunting, when given other covariates were controlled for. For children whose household had intermediate water, sanitation, and hygiene practices, the odds of being stunted were 1.01 (95% CI 0.94-1.09) times those of children in household with the lowest level water, sanitation, and hygiene practices. For children whose household had the highest versus the lowest water, sanitation, and hygiene practices level, the odds ratio of being stunted was estimated to be 0.41 (95% CI 0.18-0.85).

The estimated coefficients of total effects and average causal mediation effects (Table 3) can be interpreted as the increased or decreased risk of stunting among children whose mothers have ever been exposed to a type of mass media compared with those whose mothers have never been exposed to this type of mass media. Mothers' exposure to magazines or newspapers had no significant total effect on stunting (*P*=.75). In comparison, mothers’ exposure to any of the other 3 types of media had significantly positive effects on decreasing the risk of child stunting, with 2% (*P*=.02), 5% (*P*<.001), and 10% (*P*<.001) decreased risk of stunting for radio, television, and internet, respectively. These total effects from the mediation analysis package are consistent with the total effects from logistic regression (model 1). For mothers’ exposure to television or internet, there was a significant effect on decreasing stunting risk mediated by the composite water, sanitation, and hygiene variable, indicating a partial mediation effect with proportions of mediation of 18% (*P*=.03) and 19% (*P*=.02), respectively.

**Table 1:** Characteristics of the study participants by exposure to mass media.

Characteristics			Total (n=19,397)		Television (n=2300)		Magazine or newspaper (n=1084)		Radio (n=3943)		Internet (n=342)
Stunting, n (%)			8619 (44.43)		627 (27.26)		374 (34.50)		1468 (37.23)		74 (21.64)
Mother’s age (years), mean (SD)			29.99 (7.17)		29.74 (6.72)		29.41 (7.13)		29.89 (6.99)		28.90 (6.12)
**Household water, sanitation, and hygiene, n (%)**											
	Improved drinking water source	7109 (36.65)		1838 (79.91)		599 (55.26)		2085 (52.88)		284 (83.04)	
	Appropriate point-of-use water treatment	426 (2.20)		201 (8.74)		70 (6.46)		174 (4.41)		68 (19.88)	
	Improved toilet	2184 (11.26)		440 (19.13)		165 (15.22)		621 (15.75)		90 (26.32)	
	Appropriate handwashing practice	2267 (11.69)		700 (30.43)		282 (26.01)		826 (20.95)		151 (44.15)	
**Child’s age (years), n (%)**											
	0	4175 (21.52)		450 (19.57)		224 (20.66)		839 (21.28)		76 (22.22)	
	1	3945 (20.34)		503 (21.87)		241 (22.23)		830 (21.05)		76 (22.22)	
	2	3764 (19.41)		462 (20.09)		212 (19.56)		753 (19.10)		65 (19.01)	
	3	3914 (20.18)		464 (20.17)		227 (20.94)		775 (19.66)		69 (20.18)	
	4	3599 (18.55)		421 (18.30)		180 (16.61)		746 (18.92)		56 (16.37)	
**Mother's education, n (%)**											
	Below primary school or no formal schooling	4480 (23.10)		115 (5.00)		48 (4.43)		429 (10.88)		13 (3.80)	
	Primary school	7672 (39.55)		346 (15.04)		128 (11.81)		1207 (30.61)		41 (11.99)	
	Secondary and above	7245 (37.35)		1839 (79.96)		908 (83.76)		2307 (58.51)		288 (84.21)	
**Family wealth, n (%)**											
	Low	5859 (30.21)		220 (9.57)		224 (20.66)		816 (20.69)		45 (13.16)	
	Low-middle	4394 (22.65)		280 (12.17)		226 (20.85)		782 (19.83)		45 (13.16)	
	Middle	3759 (9.38)		548 (23.83)		224 (20.66)		767 (19.45)		45 (13.16)	
	High-middle	3023 (15.58)		636 (27.65)		203 (18.73)		781 (19.81)		74 (21.64)	
	High	2362 (12.18)		616 (26.78)		207 (19.10)		797 (20.21)		133 (38.89)	
**Child sex, n (%)**											
	Male	9564 (49.31)		1132 (49.22)		523 (48.25)		1939 (49.18)		187 (54.68)	
	Female	9833 (50.69)		1168 (50.78)		561 (51.75)		2004 (50.82)		155 (45.32)	
**Residency, n (%)**											
	Urban	5206 (26.84)		1879 (81.70)		605 (55.81)		1903 (48.26)		274 (80.12)	
	Rural	14191 (73.16)		421 (18.30)		479 (44.19)		2040 (51.74)		68 (19.88)	

**Table 2:** Effects of mothers' exposure to mass media on the association between stunting and water, sanitation, and hygiene.

Media type	Model 1: Total effects on stunting			Model 2: Effects on water, sanitation, and hygiene			Model 3: Direct effects on stunting	
	OR^a^ (95% CI)	*P* value	OR (95% CI)		*P* value	OR (95% CI)		*P* value
Magazine or newspaper	1.01 (0.87, 1.17)	.90	0.91 (0.76, 1.08)		.28	1.01 (0.88, 1.17)		.86
Radio	0.90 (0.82, 0.98)	.01	0.99 (0.90, 1.10)		.88	0.89 (0.82, 0.98)		.01
Television	0.78 (0.69, 0.89)	<.001	1.64 (1.40, 1.93)		<.001	0.79 (0.69, 0.90)		<.001
Internet	0.60 (0.45, 0.79)	<.001	2.89 (2.02, 4.12)		<.001	0.63 (0.47, 0.82)		<.001

^a^OR: odds ratio.

**Table 3:** The mediating effect of the composite water, sanitation, and hygiene variable on mothers’ exposure to mass media and stunting.

Media type	Total effect			Average causal mediation effect			Proportion of mediation^a^	
	RD^b^ (95% CI)	*P* value	RD (95% CI)		*P* value	RD (95% CI)		*P* value
Magazine or newspaper	0.00 (–0.02, 0.03)	.75	0.00 ( –0.00, 0.01)		.28	0.05 ( –1.95, 1.68)		.76
Radio	–0.02 (–0.04, –0.00)	.02	0.00 (–0.00, 0.00)		.92	–0.00 (–0.21, 0.12)		.92
Television	–0.05 (–0.08, –0.03)	<.001	–0.01 (–0.02, –0.00)		.03	0.18 (0.02, 0.39)		.03
Internet	–0.10 (–0.14, –0.06)	<.001	–0.02 (–0.03, –0.00)		.02	0.19 (0.03, 0.42)		.02

^a^Proportion of mediation = Average causal mediation effect / Total effect.

^b^RD: risk difference.

## Discussion

In the Democratic Republic of Congo, we found that mothers' exposure to television and the internet could significantly decrease their children's risk of having stunted growth through the mediation effect of the composite water, sanitation, and hygiene practices variable. In addition, we also found that mothers’ exposure to television and the internet could increase their household water, sanitation, and hygiene practices, and that children with better household water, sanitation, and hygiene have a lower risk of stunting. To the best of our best knowledge, this is the first pathway analysis study about the pathway from media exposure to stunting. Our analysis was conducted with a well-established mediation method in a counterfactual framework that provides a sound theoretical basis for causal inference. Moreover, we used a comprehensive range of indicators to represent water, sanitation, and hygiene facilities and practices of households in the Democratic Republic of Congo, and because the study utilizes data from a national survey, it is representative of the whole population.

Our results were consistent with those from some previous studies. Low- and middle-income countries in Asia, such as India and Bangladesh, found that children were more likely to have severely stunted growth if their mothers had never been exposed to mass media [37-40]. A plausible explanation could be that mothers are able to gain more information about nutrition and childcare from mass media. Moreover, other studies [24,31,41] have indicated media access is associated with water, sanitation, and hygiene–related knowledge and behaviors. Poor water, sanitation, and hygiene is related to the substantial global burden of disease and disability due to subsequent malnutrition [42,43]. For instance, improving access to water, sanitation, and hygiene can reduce the burden of infectious diseases, such as diarrheal diseases, which is associated with the risk of stunting [43,44]. Evidence shows that poor water, sanitation, and hygiene is responsible for approximately half of mothers and children who are underweight, because of the synergy, wherein one increases vulnerability to the other, between diarrheal diseases and undernutrition [45].

Given that our findings suggest that there is a positive association between media access and water, sanitation, and hygiene practice, it is possible that mass media could be used as the primary means of health intervention and health education. Published literature has discussed how mass media can be used to promote health knowledge and behaviors [46]—radios were reportedly the most frequently used channel, followed by television [24,47]. This is consistent with our results, which indicated that radio usage has the highest usage. These traditional forms of media—radio, and television—have been used to increase water, sanitation, and hygiene knowledge and behaviors among target groups [24]. In addition, we found that exposure to the internet had the largest effect on decreasing the risk of stunting. Findings from the Indonesia National Nutrition Communication Campaign revealed that the use of social media, such as Facebook, Twitter, Instagram, and YouTube, extended the campaign's reach and strengthened messaging from other sources [48]. Many campaigns also have demonstrated that the use of mass media is effective in increasing child survival [46]. In addition to water, sanitation, and hygiene–related behaviors, there were many other child survival health behaviors that mass media could impact, such as oral rehydration therapy [49], bed net use [50,51], and vaccination [52].

In addition, we found that although the internet is the most effective type of mass media to influence stunting rates through water, sanitation, and hygiene practices, it had a minor rate of use. On the other hand, radio was the most popular media type but only had a small mediation effect of water, sanitation, and hygiene practices on the association between mothers' exposure to mass media and the children's stunting status. This may be partly attributed to the convenience of receiving information from the internet for mothers as individuals [53]. Instead of sharing the radio or television channels with other family numbers, women can access health information on their own through the internet (eg, child health care and feeding). Moreover, unlike watching television and reading newspapers, new media technologies are more interactive, which allows more engagement [54]. For example, many websites and apps employ a computer algorithm to target users by their preference. By searching or reading nutrition-related pages on the internet, users are prompted with similar articles by websites. Conventional mass media do not share these characteristics. The Democratic Republic of Congo WASH Consortium also reported that the visits to its website had a higher rate of use than those of other communication tools [21]. In addition, exposure to magazines or newspapers was not significant in our model (*P*=.28). The reason for this might be the higher costs of and consequently limited access to magazines and newspapers compared with the convenience of website and social media [55]. However, to identify why the internet is more effective than traditional methods of mass media in decreasing stunting risk, additional studies are needed in order to obtain more data. Meanwhile, it might increase information disparity if any campaign focuses only on the internet, because the internet access rate is low on average but relatively higher among the households with high socioeconomic status.

Our study also had some limitations. First, data were from a cross-sectional study [33] whose observational nature without temporal sequence makes it challenging to make strong causal claims. Although we controlled for potential confounders measured in our data sets, there still could exist unmeasured exposure–outcome, exposure–mediator, and mediator–outcome confounders. For instance, paternal factors were not considered in the models due to the limitation of the MICSs data set. As a result, we were unable to differentiate the effects of maternal exposure to mass media from those of paternal source. Therefore, our exposure of interest in this study should be interpreted as an approximation of the household’s exposure to mass media rather than strictly maternal exposure. Second, we did not control for the increase in familywise error rate for multiple statistical tests. Overall, we consider this study to be preliminary and encourage further investigation in this field. Lastly, mediation model warrants further refinement through interdisciplinary collaboration on questions such as whether and how information not directly related to water, sanitation, and hygiene in mass media can affect household water, sanitation, and hygiene practices.

We used causal mediation analysis to reveal the pathway from media exposure to stunting status in the Democratic Republic of Congo. As stunting in children continues to be a severe issue in the Democratic Republic of Congo, the findings of this study can inform and guide interventions or policies to reduce the rate of stunting among children by promoting water, sanitation, and hygiene through mass media. Mass media campaigns, using internet and television, may improve water, sanitation, and hygiene practices and help prevent stunting.

## Appendix

Multimedia Appendix 1 Characteristics of the study participants by exposure to mass media.

## Abbreviations

MICS: Multiple Indicators Cluster Surveys
WASH: Water, Sanitation, and Hygiene program
WHO: World Health Organization
UNICEF: United Nation Children’s Fund
