# Supplementary material for: Influence of Maternal Exposure to Mass Media on Growth Stunting Among Children Under Five: Mediation Analysis Through the Water, Sanitation, and Hygiene Program
Source: JMIR Public Health Surveill. 2022 Apr 6;8(4):e33394. doi: 10.2196/33394 (PMC9021946; doi:10.2196/33394)
Supplement: Multimedia Appendix 1 [file publichealth_v8i4e33394_app1.docx]

| Appendix 1. CHARACTERISTICS OF THE STUDY PARTICIPANTS BY EXPOSURE TO MASS MEDIA | | | | | |
| --- | --- | --- | --- | --- | --- |
|  | Total | Children whose mother had ever watched television | Children whose mother has ever read magazine or newspaper | Children whose mother has ever listened to the radio | Children whose mother has ever used internet |
| N | 19397 | 2300 | 1084 | 3943 | 342 |
| Stunting(%) | 44.43% | 27.26% | 34.50% | 37.23% | 21.64% |
| Province(%) |  |  |  |  |  |
| Kinshasa | 3.09% | 21.74% | 5.72% | 4.67% | 25.15% |
| Kongo Central | 2.53% | 5.78% | 1.66% | 4.08% | 3.80% |
| Kwango | 4.38% | 4.83% | 7.75% | 6.04% | 2.34% |
| Kwilu | 3.12% | 2.48% | 1.11% | 1.55% | 0.00% |
| Maindombe | 3.14% | 1.39% | 2.21% | 1.62% | 3.22% |
| Equateur | 3.54% | 2.39% | 1.85% | 2.36% | 2.05% |
| Sud Ubangi | 4.26% | 1.26% | 4.34% | 7.10% | 1.17% |
| Nord Ubangi | 4.19% | 1.70% | 3.97% | 3.83% | 1.46% |
| Mongala | 3.61% | 1.83% | 7.66% | 4.72% | 1.46% |
| Tshuapa | 3.72% | 2.91% | 4.34% | 1.83% | 2.63% |
| Tshopo | 4.14% | 5.52% | 6.18% | 4.89% | 5.26% |
| Bas Uele | 3.10% | 0.61% | 2.86% | 3.09% | 2.05% |
| Haut Uele | 2.77% | 0.78% | 5.35% | 3.14% | 0.58% |
| Ituri | 3.51% | 0.57% | 2.77% | 3.93% | 1.75% |
| Nord Kivu | 4.13% | 4.30% | 2.40% | 5.48% | 12.87% |
| Sud Kivu | 5.05% | 3.04% | 3.60% | 4.16% | 5.26% |
| Maniema | 3.79% | 0.83% | 1.57% | 0.81% | 0.88% |
| Haut Katanga | 4.35% | 19.00% | 8.67% | 9.56% | .28% |
| Lualaba | 3.75% | 9.39% | 2.68% | 3.93% | 3.22% |
| Haut Lomami | 4.86% | 1.57% | 1.66% | 5.05% | 2.05% |
| Tanganyika | 3.18% | 1.09% | 2.49% | 1.17% | 1.17% |
| Lomami | 4.46% | 0.52% | 4.15% | 2.46% | 0.29% |
| Kasai Oriental | 4.23% | 4.04% | 9.78% | 5.43% | 6.14% |
| Sankuru | 4.49% | 1.87% | 1.57% | 4.92% | 0.00% |
| Kasai Central | 4.20% | 0.26% | 2.31% | 2.41% | 0.58% |
| Kasai | 4.41% | 0.30% | 1.38% | 1.78% | 2.34% |
